# Lysophosphatidic Acid: Promoter of Cancer Progression and of Tumor Microenvironment Development. A Promising Target for Anticancer Therapies?

**DOI:** 10.3390/cells10061390

**Published:** 2021-06-04

**Authors:** Sistiana Aiello, Federica Casiraghi

**Affiliations:** Istituto di Ricerche Farmacologiche Mario Negri IRCCS, 24126 Bergamo, Italy; federica.casiraghi@marionegri.it

**Keywords:** lysophosphatidic acid, autotaxin, cancer, tumor microenvironment, fibrosis, immune escape

## Abstract

Increased expression of the enzyme autotaxin (ATX) and the consequently increased levels of its product, lysophosphatidic acid (LPA), have been reported in several primary tumors. The role of LPA as a direct modulator of tumor cell functions—motility, invasion and migration capabilities as well as resistance to apoptotic death—has been recognized by numerous studies over the last two decades. Notably, evidence has recently been accumulating that shows that LPA also contributes to the development of the tumor microenvironment (TME). Indeed, LPA plays a crucial role in inducing angiogenesis and lymphangiogenesis, triggering cellular glycolytic shift and stimulating intratumoral fibrosis. In addition, LPA helps tumoral cells to escape immune surveillance. Treatments that counter the TME components, in order to deprive cancer cells of their crucial support, have been emerging among the promising new anticancer therapies. This review aims to summarize the latest knowledge on how LPA influences both tumor cell functions and the TME by regulating the activity of its different elements, highlighting why and how LPA is worth considering as a molecular target for new anticancer therapies.

## 1. Introduction

Lysophosphatidic acid (LPA) is a robust extracellular signaling molecule that regulates various cellular processes, such as migration, proliferation and survival. Most LPA is generated by the enzyme autotaxin (ATX). Over the past two decades, a large body of literature has indicated that the ATX/LPA axis plays a pivotal role in the pathogenesis of fibrosis in several organs, including the lung [1,2], kidney [3,4], liver [5] and skin [6]. Thus, in search of new antifibrotic therapies, inhibition of either the ATX activity or the LPA signaling pathway has been tested in various experimental models of fibrotic diseases. In this regard, in an experimental model of chronic allograft nephropathy characterized by severe kidney graft fibrosis, we reported that treatment with an orally available ATX inhibitor strongly limited renal fibrosis and significantly prolonged the survival of allotransplanted animals [7]. Similarly, blocking the LPA in a murine lung transplant model of bronchiolitis obliterans syndrome abated allograft fibrosis, as demonstrated by morphometric analysis of airway remodeling and collagen deposition [8].

Fibrosis is defined as tissue overgrowth and hardening due to an excess deposition of extracellular matrix components and is a characteristic hallmark of several clinical entities, including chronic kidney diseases, scleroderma, idiopathic pulmonary fibrosis and cancer. In the context of cancer, fibrotic/desmoplastic lesions often accompany tumor development and progression, and their presence is correlated with the patient poor prognosis.

Numerous pieces of evidence showing a role of LPA in promoting several functions and activities of tumor cells have been accumulated over the last three decades and have been extensively described in dedicated recent reviews [9,10,11]. This review has been constructed with the aim of exploring the role of the ATX/LPA axis in tumor development and progression with particular attention to the role of the ATX/LPA axis as a promoter of fibrosis and tumor microenvironment development (TME). In addition, since very recent data documented the success of phase II clinical trials with ATX/LPA blockers in patients with idiopathic pulmonary fibrosis (IPF) [12,13], the aim of the review is also to explore how LPA is worth considering as a molecular target for anticancer therapies or even for adjuvant therapies.

## 2. Autotaxin, LPA and Its Receptors

LPA is a small glycerophospholipid consisting of an acyl chain at the sn-1 (or sn-2) position of a glycerol backbone and a phosphate head group [14,15]. Depending on the length and saturation of the acyl or alkyl fatty acid chain, several LPA species can be found.

LPA is generated by the enzyme ATX (the gene name is *ENPP2*) starting from membrane lysophospholipids or from circulating lysophosphatidylcholine bound to albumin [16]. ATX is a widely expressed 125kDa-secreted enzyme from the family of ectonucleotide pyrophosphatases/phosphodiesterases [17]. Among the seven members of this family, ATX is a unique enzyme showing lysophospholipase D activity [18]. Some LPA, primarily the saturated species, can also be generated from the substrate phosphatidic acid through the catalytic activity of the soluble form of phospholipase A_2_ [18]. However, most of the extracellular LPA is generated by ATX, since mice with the heterozygous loss of ATX exhibit plasmatic levels of LPA that are half as high as normal [19]. LPA is turned over rapidly (t_1/2_ of around 1 min) in plasma by the ecto-activity of three lipid phosphate phosphatases (LPP1-3) [16,20]. This ecto-activity helps regulate the total LPA pool and, in particular, reduces LPA concentrations in the immediate cellular microenvironment [21]. Recently, Benesch et al. described a negative feedback loop of the ATX/LPA axis that is crucial to further control the LPA concentrations [22]. In this mechanism, when LPA accumulates in the circulation, it induces the downregulation of ATX mRNA [22], whereas physiological concentrations of LPA have little inhibitory effect on ATX activity.

LPA is present in all eukaryotic tissues and blood plasma [23,24] under both physiologic and pathologic conditions [25]. This bioactive phospholipid plays a crucial role in ensuring proper organism development. This is especially evident from mouse models, as either ATX knockout or overexpression is embrionically lethal at around day 9.5–11.5 due to neural crest anomalies and severe vascular defects [19,26,27]. In the postnatal organism, LPA is crucial in mediating wound healing and tissue remodeling by virtue of its capability of promoting several cellular activities and functions, including cell proliferation, migration, survival and cytoskeletal reorganization [28,29,30]. Within the damaged tissue, inflammatory cytokines—such as TNF-α and IL-1β—can stimulate the ATX activity, thus leading to the increased levels of LPA necessary for repair processes [31]. If the healing process is successful and inflammation subsides, the ATX-produced LPA feeds back and blocks further ATX, as above described in the study of Benesch et al. [22]. However, if the healing process is not properly resolved, the LPA–ATX negative feedback loop can be overcome by the presence of excessive inflammatory cytokines that affect the capability of LPA to block ATX activity [22], and thus an inflammatory vicious cycle can occur. This situation particularly applies to cancer, which has been often described as “a wound that never heals” [32,33,34], giving a possible explanation for the high levels of ATX and LPA documented in cancers.

LPA exerts its activities by acting through specific plasma membrane-associated LPA receptors. LPA receptors belong to the family of the G protein-coupled receptors and signal through activation of the G_i_, G_s_, G_q_ or G_12/13_ subunit [25]. Starting with the first LPA receptor, which was identified and cloned in 1996 [35], a total of six specific LPA receptors have been described, namely LPA_1-6_ [36]. LPA can also be synthesized intracellularly. In this case, the intracellular LPA receptor is the nuclear receptor peroxisome proliferator-activated receptor gamma (PPARγ) [37].

LPA_1–6_ are expressed differently in body tissues and organs. LPA_1_ is widely expressed in several organs, including the brain, lung, heart, kidney, spleen, small intestine, stomach, uterus and placenta [29,30,38]. On the other hand, the other LPA receptors exhibit relatively organ-restricted expression [38]. For example, high levels of LPA_2_ have been found in leukocytes, testis, kidney and uterus; LPA_3_ expression is most prominent in heart, prostate and pancreas; and LPA_4_ is mainly expressed in ovary [29,38].

The cell/tissue-specific expression of the different LPA receptors enables the different cancer cells to respond to LPA stimulation in a unique manner and explains the diversification of LPA-induced biological activities, even on the same cellular type [25]. Hama et al., through the examination of the expression of LPA_1_, LPA_2_, LPA_3_ and LPA_4_ in different cancer cell lines, found that (1) the various cancer cells express LPA receptors differentially and (2) only the cells expressing LPA_1_ showed LPA-dependent motility [39]. In detail, the cancer cell lines expressing moderate to high levels of LPA_1_, such as MDA-MB-231 (breast cancer), PC-3 (prostate cancer), A-2058 (melanoma), A549 (lung cancer), ACHN (renal cancer) and SF295 (glioblastoma), exhibited enhanced migration in response to LPA; however, LPA did not support the migration of MCF7 (breast cancer), KM-12 (colorectal cancer) and OVCAR-4 (ovarian cancer), which express LPA_2_ but not LPA_1_, or the migration of LNCaP (prostate cancer), which expresses LPA_3_ but not LPA_1_ [39]. On the other hand, by using a transgenic neuroblastoma cell line expressing LPA_1_, LPA_2_ or LPA_3_, Hayashi et al. documented that neuroblastoma cells expressing LPA_2_ and LPA_3_, but not those expressing LPA_1_, showed increased cell motile and invasive activities in response to LPA [40].

Those pieces of evidence would indicate that the tumor cell response to LPA is a complex issue since it can vary depending on several factors, including the cancer cell type stimulated, the tumor stage, the LPA receptor expressed and the LPA species.

## 3. LPA: Direct Effects on Tumor Initiation and Progression

An increase in the expression of ATX, originally identified in 1992 as an “autocrine motility factor” secreted by melanoma cells [41], has been found in several human cancers [42,43,44,45,46,47,48]. As a consequence of the increased ATX expression, tumors are characterized by LPA levels that are higher than in normal tissues [49,50]. The first report documenting an association between LPA and cancer was published in 1995. Xu and coworkers showed that LPA was present at elevated levels in ascites of patients with ovarian cancers. They also documented that, in vitro, LPA was able to activate the proliferation of both ovarian and breast cancer cells through a combination of signals, including increases in intracellular [Ca^2+^], tyrosine phosphorylation and MAP kinase activation [51].

Subsequent studies revealed that the ATX/LPA axis had direct effects on the initiation and progression of several types of tumor, as detailed in the following paragraphs.

### 3.1. ATX/LPA Axis: A Promoter of Tumorigenesis, Tumor Invasion and Metastasis

A remarkable study by Liu et al. indicated that the ATX/LPA axis might have direct oncogenic effects on normal cells. Indeed, by using transgenic mouse models that express either ATX or one of the LPA receptors—LPA_1_, LPA_2_ and LPA_3_—under the mouse mammary tumor virus-long terminal repeat (MMTV-LTR) promoter, the authors demonstrated that forced expression of ATX or of one of the three LPA receptors in the mammary glands was sufficient to induce the spontaneous development of breast cancer [52]. On the other hand, in a model of cancer development induced by carcinogenic compounds, tumorigenesis is associated with the modulation of the expression of different LPA receptors. Indeed, in pancreatic duct adenocarcinoma (PDAC) induced in hamsters using the nitroso compound N-nitrosobis(2-oxopropyl)amine, mRNA levels of LPA_1_ and LPA_3_ were found lower and higher, respectively, than in normal pancreatic tissue. This finding led the authors to hypothesize that the downregulation of LPA_1_ and upregulation of LPA_3_ were associated with the occurrence of the tumorigenic phenotype in the pancreatic duct cells [53].

Further studies in mice have shown that the autocrine activity of the ATX/LPA axis is also capable of enhancing tumor aggressiveness. Indeed, ATX-transfected/Ras-transformed NIH-3T3 cells injected into nude mice have been shown to be more tumorigenic, invasive and metastatic than Ras-transformed control cells [54]. Moreover, in mouse PDAC, Auciello et al. reported that genetic disruption of *Enpp2* (the gene encoding ATX) strongly reduced the in vitro proliferative capability of PDAC cells. In addition, compared with immune-competent mice that received control PDAC cells in the pancreas, mice that received *Enpp2* ko PDAC cells exhibited compromised tumor growth [55].

In addition to acting as an oncogenic driver or enhancer of tumor aggressiveness, the ATX/LPA axis also helps tumor progression. Indeed, many experimental studies with cancer cell lines or experimental models have revealed that LPA signaling plays a crucial role in cancer cell proliferation and growth, as well as in motility and invasiveness [39,40,46,56,57]. LPA-induced effects can be various depending on the cancer cell type studied and the LPA receptor expressed. For example, LPA_1_ and LPA_3_ had opposite effects on cell motility and the invasive activities of pancreatic cancer cells [53]. Indeed, proliferative, motile and invasive capacities were defective in LPA_3_-deficient hamster PDAC cells, whereas those capacities were enhanced in LPA_1_-deficient PDAC cells. On the other hand, in human ovarian cancer cells, proliferation was dependent on signaling through LPA_2_ and LPA_3_ but not LPA_1_, whereas motility and invasion were mediated by signaling through LPA_1_, LPA_2_ and LPA_3_ [57].

The mechanisms underlying LPA-induced cancer cell invasiveness are well studied in liver cancers. Hepatocellular carcinoma (HCC) is a type of primary hepatic carcinoma that accounts for 90% of primary liver cancers. Unlike in the normal liver, in HCC, ATX, LPA and LPA_1_ are highly expressed. The inhibition of LPA_1_ together with the inhibition of the phosphoinositide 3-kinase (PI3K)/Akt and protein kinase Cδ (PKCδ)/p38-MAPK pathways all result in decreased MMP-9 activity and invasiveness of HCC, indicating that LPA enhances MMP-9 expression and HCC invasiveness through the LPA_1_ receptor and synergistic activation of the PI3K and p38-MPAK signaling cascades [58,59].

The ATX/LPA axis also has prometastatic properties. Various studies have shown that the LPA-induced invasive properties can be different, possibly depending on the cancer cell type stimulated, the tumor stage and the LPA receptor engaged. In the model of spontaneous development of breast cancer in LPA_1–3_-transgenic MMTV-LTR mice, the forced expression of LPA_1_, LPA_2_ or LPA_3_ resulted in an increased occurrence of invasive and metastatic mammary tumors. The rate of breast cancer metastases was significantly higher in LPA_3_ transgenic mice than in those overexpressing LPA_1_ or LPA_2_ [52]. Accordingly, by analyzing the expression of ATX and LPA_3_ in 87 invasive human breast carcinomas, Popnikolov et al. demonstrated that (1) compared with normal breast tissue, mammary carcinomas were more frequently positive for ATX and LPA_3_, and (2) compared with LPA_3_^−^ tumors, the LPA_3_-expressing tumors presented with a more advanced stage of disease and more often had lymph node metastases [60].

On the other hand, tumor metastatic capability was mediated mainly by LPA_1_ signaling in another model of breast cancer. In the breast cancer model induced in immune-competent Balb/c mice through intramammary injection of murine 4T1 mammary carcinoma cells, tumor capability to metastasize early was mediated mainly by LPA_1_, and the administration of a highly specific LPA_1_ antagonist, Debio-0719, reduced the number of spontaneously disseminated tumor cells to bones and the lungs [61]. In breast cancer patients, authors found that augmented LPA_1_ mRNA expression in primary tumors correlated with their positive lymph node status [61].

At the clinical level, serum levels of LPA may represent a marker of tumor progression severity. Indeed, Mazzocca et al. found that LPA serum levels were higher in HCC than in healthy controls or liver cirrhosis patients [62] and, among HCC patients, LPA serum levels were higher in those with metastasis compared to those without. Moreover, patients with higher serum levels of LPA also have larger HCC tumors and shorter survival compared with those with lower LPA serum concentrations [62].

### 3.2. ATX/LPA Axis: A Promoter of Tumor Cell Survival and Antitumor Therapy Resistance

In the transgenic mice that overexpress ATX or LPA_1-3_ under the MMTV-LTR promoter [52], the spontaneous development of mammary tumors displayed late onset, ranging from 8 to 24 months, and occurred in a subpopulation of each transgenic mouse strain, suggesting that expression of ATX and LPA receptors cooperates with other events, such as secondary mutations, to generate the full tumorigenic phenotype [52]. The mechanism proposed was based on the capability of LPA to enhance cell survival, which allows secondary mutations to be “fixed” in the cell genome [52].

Indeed, LPA has been recognized as a potent survival factor that promotes tumor cell survival under stress conditions, such as those caused in vitro by serum withdrawal [49] or by excessive DNA damage induced by UV light [57]. In normal cells, the activation of the p53 protein promotes the G_1_–S cell cycle arrest and/or apoptosis in response to DNA damage [63]. Accordingly, 50% of all cancers exhibited mutated or inactivated p53, which enhances the ability of cancer cells to evade cell cycle arrest or apoptosis. Remarkably, a study by Murph et al. revealed that LPA, via LPA_1–3_ engagement and intracellular activation of the PI3K/Akt signaling pathway, inhibits p53 activity and protects tumor cells from actinomycin D-induced apoptosis [64], indicating that LPA is potentially an inducer of drug resistance in tumors. Further studies have shown that ovarian cancer cells that express high LPA_1_ levels are more resistant to cisplatin-induced apoptosis [65], while LPA_3_ expression in hepatocarcinoma and breast cancer cells favors cell survival under cisplatin and doxorubicin treatments [66]. In addition, in MCF-7 breast cancer cells, Samadi et al. showed that LPA partially reversed the Taxol-induced G_2_–M cell cycle arrest and strongly antagonized Taxol-induced apoptosis through the stimulation of PI3K and the inhibition of ceramide formation [67]. More recently, LPA has been shown to protect tumor cells against apoptosis induced by radiotherapy or chemotherapy, mainly by interacting with LPA_2_, which results in depleting the cell of Siva-1 (a proapoptotic signaling protein) and in stimulating prosurvival pathways through a TRIP-6-mediated mechanism [68].

These findings clearly indicate that LPA is a prosurvival factor for tumor cells and a possible therapeutic target for overcoming tumoral resistance to anticancer therapies.

## 4. LPA: Effects on the Tumor Microenvironment

Solid tumors develop within a complex and highly heterogeneous milieu consisting of different cellular and noncellular elements, including cancer-associated fibroblasts (CAFs), endothelial cells and pericytes that compose the tumor vasculature, lymphatics, immune cells and extracellular matrix proteins [69]. The tumor microenvironment (TME) is critical to supporting tumor survival and growth, as well as the process of metastatic dissemination. The TME also helps the tumor in the process of immune evasion by blocking antitumor immunity (e.g., inhibiting T cell cytotoxic activity [70,71]) while promoting protumor immune cells, such as anti-inflammatory M2 macrophages [72], T regulatory cells [73,74] and myeloid-derived suppressor cells [75].

Beyond cancer cells, the TME is also an important source of ATX and LPA. In some cases, the TME is the primary source of ATX, as suggested by data of 4T1 mouse mammary primary tumors that exhibit higher ATX staining in the stroma than in the tumor cell compartment [76]. As analyzed in depth in the following paragraphs, LPA contributes to TME development and maintenance in several ways: by stimulating angiogenesis, promoting the metabolic glycolytic shift, generating CAFs and consolidating fibrosis, as well as by supporting tumor immune escape.

### 4.1. ATX/LPA Axis: A Promoter of Angiogenesis and Lymphangiogenesis

Angiogenesis is a process that is essential for the growth of primary tumors, the formation of the TME and the dissemination of metastases [77].

By using an experimental model of in vivo angiogenesis obtained through s.c. injection of matrigel plugs into nude mice, Nam and coworkers clearly demonstrated that ATX works as a proangiogenic factor. The study showed that mixing ATX-transfected/Ras-transformed NIH-3T3 cells into the matrigel resulted in greater formation of new blood vessels than mixing control cells [78]. Similarly, mixing purified ATX alone into matrigel resulted in the formation of new blood vessels within the plug, which was comparable to that induced by vascular endothelial growth factor (VEGF) [78], the most potent angiogenic factor produced by cancer cells [79].

Studies that report that LPA/LPA_2-3_ engagement stimulates VEGF production in ovarian cancer cells [57,80] indicate that the LPA proangiogenic mechanism is based on the stimulation of intratumoral release of VEGF. At the clinical level, this is supported by results that demonstrate a positive correlation between LPA_2-3_ and VEGF expression in human ovarian cancer biopsies [81]. However, these findings cannot be extended to all types of cancers. In vitro experiments with several breast cancer cell lines, which are known to release high amounts of VEGF [82], have shown that breast cancer cells do not release further VEGF in response to LPA stimulation [83]. LPA-induced angiogenic effects can also be mediated by CAFs. Indeed, cancer-derived LPA stimulated CAFs to secrete proangiogenic factors, such as SDF-1α and VEGF [84].

Many tumors, including prostate cancer (PCa), first metastasize to regional lymph nodes via lymphatic vessels. Thus, beyond angiogenesis, lymphangiogenesis is also an important mechanism of metastasis establishment. The major lymphangiogenic factor is VEGF-C, a member of the VEGF protein family, which activates lymphangiogenesis-associated signaling pathways by binding to VEGFR-3 [85]. In PCa, antagonizing VEGFR-3 has been reported to be an effective form of anticancer therapy, as suggested by results showing that a specific VEGFR-3 antagonist successfully inhibits lymphangiogenesis and cancer metastasis in PCa-bearing mice [86]. LPA has been documented to promote lymphangiogenesis through a VEGF-C-dependent mechanism. Indeed, binding of LPA to LPA_1_ and LPA_3_ stimulated VEGF-C transcription and production in human prostate cancer PC-3 cells [87]. Notably, in patients’ PCa biopsies, high expression levels of ATX and VEGF-C have been associated with a high density of lymphatic vessels and the severity of PCa (Gleason score >6) [88]. Importantly, PCa-bearing nude mice treated with Ki16425—a LPA_1_/LPA_3_ pharmacological antagonist—exhibited a significant reduction in the intratumor lymphatic vessel density and lymph node metastasis rate, compared with PCa-bearing mice treated with vehicle [88].

Thus, by antagonizing the process of angiogenesis/lymphangiogenesis, the blockade of the ATX/LPA axis could offer a new target to be exploited to halt tumor progression.

### 4.2. ATX/LPA Axis: A Promoter of Tumor Glycolytic Shift

For decades, hypoxia and acidosis have been described as two ubiquitous features of the TME [89]. Within the TME, the continuous outgrowth of cancer cells, accompanied by improper angiogenesis and insufficient oxygen supply, is the main cause of hypoxia, which correlates with cancer progression and poor prognosis [90]. Hypoxia-induced oxidative stress, through the stimulation of hypoxia-inducible factor 1 α (HIF1α), has been shown to play a major role in reprogramming the cancer cell metabolism toward enhanced glycolysis and elevated lactate metabolism, a phenomenon named the Warburg effect, which is eventually responsible for extracellular H^+^ accumulation and intratumoral acidosis. Intratumoral acidosis in turn plays a key role in maintaining the TME and advancing cancer progression, since it increases the epithelial–mesenchymal transition, promotes tumor invasion and metastasis and has regulative effects on immune cells (e.g., by polarizing macrophages toward the anti-inflammatory M2 phenotype and inhibiting effector T cell functions) [89,91].

LPA has recently been included among the extrinsic stimuli that can induce cancer metabolic reprogramming towards the glycolytic shift. Ha and collaborators evaluated the proglycolytic effect of LPA on a panel of ovarian cancer cell lines with different genetic backgrounds and subtypes. In all the ovarian cancer cell lines tested, LPA induced an increase in both the rate of glycolysis and glycolytic capability [92]. The mechanism involved (1) linking LPA to an LPA receptor associated with a G_αi2_ subunit; (2) activating the Rac-NOX-ROS pathway, leading to an increase in HIF1α levels; and (3) HIF1α-induced expression of the glucose transporter-1 (GLUT1) and the glycolytic enzyme hexokinase-2 (HKII). Importantly, the inhibition of HKII in mice with an ovarian cancer xenograft attenuated tumor growth and prolonged survival [92].

LPA induced the metabolic reprogramming towards glycolytic shift also in CAFs [93]. Indeed, Radhakrishnan et al. observed that LPA treatment of CAFs isolated from ovarian cancers increased both the rate of glycolysis and their glycolytic capability. The LPA-induced glycolytic shift increased in a dose-dependent manner and was prevented when CAFs were pretreated with the LPA_1/3_ antagonist Ki16425. Notably, the study showed that conditioned medium from ovarian cancer cell cultures or ascitic fluid from an ovarian cancer patient was also able to reprogram the CAF metabolism towards the glycolytic shift. The ascitic fluid-induced glycolytic shift required the engagement of LPA_1/3_ and the intracellular activity of HIF1α. Indeed, it was prevented by cotreating CAFs with the LPA_1/3_ antagonist Ki16425, as well as by silencing the expression of HIF1α in CAFs through siRNA [93].

### 4.3. ATX/LPA Axis: A Promoter of CAF Generation and Fibrosis Development

CAFs are heterogeneous cells that express myofibroblast markers, such as αSMA, vimentin, fibronectin, fibroblast-specific protein 1 (FSP-1) and fibroblast activation protein (FAP) [94]. CAFs—apart from being crucial for the synthesis of collagens, fibronectin and other TME structural components [95]—support tumor invasion and dissemination (e.g., by releasing MMP-2 and MMP-9), help cancer cell proliferation (e.g., through the release of SDF-1, FGF and osteopontin [96,97]) and promote angiogenesis (e.g., through the expression of adrenomedullin and VEGF [84,98]). In a xenograft model of HCC, coinjection of Huh7 cells with CAFs resulted in earlier development and larger tumors than those that resulted from the injection of Huh7 cells alone [62].

CAFs can originate from the transformation of stellate cells or cancer-associated adipocytes, from both epithelial–mesenchymal transition and endothelial–mesenchymal transition, as well as from the activation/differentiation of resident fibroblasts by TGF-β, PDGF or FGF-2 [96,99,100,101]. Several studies have reported that LPA is a CAF-generating factor. In vitro stimulation of human normal fibroblasts with LPA (as well as with conditioned medium from an ovarian cancer cell line) converted these cells into CAFs that expressed αSMA, TGFβ1, TGFβ2, VEGFA, VEGFB, FAP, CXCL12 and IL-6 [93]. In addition, Jeon and coworkers demonstrated that conditioned medium from ovarian cancer cell lines induced the differentiation of human adipose tissue-derived mesenchymal stem cells (hASCs) to αSMA^+^ SDF-1α^+^ VEGF^+^ CAFs [84]. Pretreating hASCs with the LPA_1/3_ antagonist Ki16425 or using short hairpin RNA lentiviral silencing on the LPA_1_ inhibited CAF generation induced by conditioned medium. LPA-mediated CAF generation required the activation of multiple signaling pathways involving Rho-kinase, ERK, PLC and phosphoinositide-3-kinase [84].

More importantly, the study by Mazzocca et al. demonstrated that tumor-derived LPA is the mediator of paracrine cross-talk between tumor cells and normal stromal fibroblasts that leads to the generation of CAFs [62]. The authors isolated cancer cells and peritumoral tissue fibroblasts (PTFs) from HCC from different patients and carried out coculture experiments in vitro and in vivo. In vitro results showed that (1) HCC cells recruit PTFs and promote their differentiation to a CAF-like αSMA^+^ myofibroblastic phenotype; (2) silencing ATX in HCC cells or inhibiting LPA using a pan-LPA inhibitor prevented PTF transdifferentiation; and (3) transdifferentiated PTFs enhanced the proliferation, migration and invasion of HCC cells. In vivo, PTFs coinjected with HCC cells in nude mice underwent transdifferentiation, promoting tumor progression. Notably, treating mice with a pan-LPA inhibitor arrested HCC growth and progression by blocking PTF differentiation to the CAF-like myofibroblastic phenotype.

Unlike normal fibroblasts, CAFs are perpetually activated and very resistant to apoptosis. During tumor progression, CAFs are the major players in the dysregulated collagen turnover leading to excessive collagen deposition (desmoplasia) and tumor fibrosis [102,103]. Within the TME, CAF-derived collagens are often crosslinked and linearized, promoting stiffening of the tumor tissue [102]. This cancer-associated fibrosis plays a central role in regulating migration, invasion and metastasis [104,105]. In addition, the accumulation of collagens, accompanied by the stiffening of the tissue, increases interstitial fluid pressure, reducing drug delivery for chemotherapy and immunotherapy and thus contributing to tumor therapy resistance [106]. Tumors characterized by a strong desmoplastic reaction and containing a large amount of CAF-derived stromal collagens are correlated with poorer patient outcomes [107,108,109,110]. Thus, the fibrotic tumor stroma is emerging as a target for cancer therapeutics.

LPA may contribute to TME fibrosis by stimulating CAFs to release ECM proteins. Indeed, lung fibroblasts, when stimulated by LPA, release elevated amounts of type I and type VI collagens, as well as fibronectin [111]. The LPA-induced effects were comparable to those observed in response to TGF-β1 and were reduced by an antagonist of LPA_1_ [111].

Since chronic fibrosis is not only a consequence but also a risk factor for cancer—as suggested by clinical evidence that IPF is an independent risk factor for lung cancer [112] or that fibrosis induced by epidermolysis bullosa is strongly associated with patient predisposition to develop squamous cell carcinoma [113]—effective antifibrotic therapy, targeting the ATX/LPA axis, could offer the advantage of combining the antifibrotic effect with preventive action against possible fibrosis-induced tumors.

### 4.4. ATX/LPA Axis: A Promoter of Tumor Escape from Immune Surveillance

To escape immune-mediated detection and eradication, tumor cells exploit different strategies, including the downregulation or loss of tumor antigens and the release of immunosuppressive molecules. The TME supports tumor cells significantly in escaping immune surveillance. Indeed, TME is an immune-hostile microenvironment that does not allow T cells to penetrate, persist and maintain their effector functions [69] and harbors large amounts of tumor-associated macrophages (TAMs), anti-inflammatory macrophages that are commonly associated with a poor prognosis [114].

Recently, it has been reported that TAMs express ATX and are the predominant source of LPA production in the ascites of ovarian cancer patients [115]. TAMs also express LPA receptors, mainly LPA_3_, LPA_5_ and LPA_6_ [115], but the role of LPA and LPA receptors in TAM generation or functions remains unknown. Based on recent reports that LPA converts circulating monocytes into macrophages [116], it would be worth exploring whether LPA is also a TAM-converting factor.

The crucial step in immune eradication of tumors is the infiltration and activation of T cells, particularly CD8^+^ cytotoxic T cells [117]. LPA may help tumor cells to counter the antitumor immune response by inhibiting the activation of CD8^+^ T cells. It has been shown that LPA inhibited intracellular calcium mobilization and ERK activity in CD8^+^ T cells in response to TCR stimulation [118,119]. Further experiments showed that (1) of the three LPA receptors expressed by CD8^+^ T cells (LPA_2_, LPA_5_, LPA_6_), LPA_5_ expression was required for the LPA-mediated inhibition of the calcium mobilization induced by TCR stimulation; (2) LPA/LPA_5_ signaling inhibits antigen-specific CD8^+^ T cell activation and proliferation, both in vitro and in vivo; and (3) LPA_5_ signaling suppresses cytotoxic activity of CD8^+^ T cells by impairing the exocytosis of granules containing granzyme B [118,119]. Notably, when mice with melanoma were treated with either WT or LPA_5_^-/-^ tumor-specific CD8^+^ T cells, high numbers of CD8^+^ T cells were found only within the tumors of mice receiving LPA_5_^-/-^ CD8^+^ T cells, and tumor growth was clearly reduced [118,119]. The hypothesis is that the engagement of LPA_5_ blocks the activation of the immunologic synapse between the CD8^+^ T cell and its target cell, thus leading to the concept that LPA_5_ functions as a novel inhibitory receptor.

However, there is evidence that the ATX/LPA axis could also help T cells to perform a proper antitumor immune response. In transwell assays, LPA has been found to induce both the chemotaxis and the chemokinesis of T cells [120,121]. Further studies have indicated that the ATX/LPA axis plays a crucial role in promoting the transmigration of T cells across high endothelial venules [122,123], suggesting that the ATX/LPA pathway is involved in the regulation of T cell trafficking from the blood into lymphoid tissues and sites of inflammation [124]. Accordingly, in a model of fully allogeneic kidney transplantation in rats, which was characterized by high amounts of intragraft-infiltrating T cells, we reported that ATX inhibition reduced allograft infiltration of CD4^+^ and CD8^+^ T cells, remarkably in the framework of the highly alloreactive environment that characterizes the kidney allotransplant model [7]. In two distinct murine models of colitis, pharmacological blockade of ATX ameliorated intestinal injury by inhibiting lymphocyte recruitment into the inflamed mucosa [125].

Since T cell trafficking is a crucial step in the immune eradication of tumors, it is very important to know whether the ATX/LPA axis drives T cell recruitment in the tumors as well and, if so, through which LPA receptor. Takeda et al. showed that LPA–LPA_2_ interaction on naïve T cells is crucial for the activation of the ROCK/myosin IIA pathway and the enhanced T cell motility and migration across very small pores [126]. Whether ATX/LPA/LPA_2_ activity promotes T cell tumor infiltration is also worth exploring.

## 5. Conclusions and Therapeutic Perspectives

**Figure 1:**
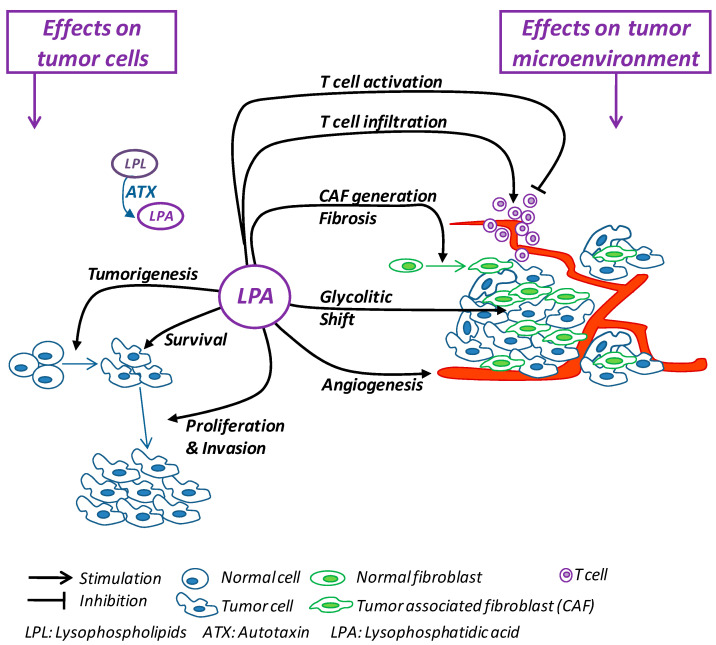
Schematic summary of the effects exerted by LPA on tumor cells and on the tumor microenvironment.

Cancer is one of the most challenging growing public health problems, so it remains imperative to develop new anticancer strategies. For decades, cancer therapy has focused on treatments aimed at countering the survival and proliferation capability of tumoral cells, mainly through chemotherapy and radiotherapy [127]. The recent evidence showing that cellular and noncellular components of the TME are crucial in helping tumor growth, invasion and metastasis, as well as in influencing the response to therapies [69], has suggested the idea of countering the components of TME to deprive cancer cells of their crucial support, switching cancer research and treatment from a cancer-centric model to a TME-centric one. As summarized in Figure 1, the numerous protumor activities carried out by LPA include direct effects on tumor cells and the promotion and maintenance of the TME. Thus, the inhibition of the ATX/LPA axis would simultaneously block tumor cells and TME components, representing a promising multitasking molecular target for building new anticancer therapies.

In search of new antifibrotic therapies, preclinical studies with anti-ATX/LPA molecules showed promising results [7,8,128]. A first encouraging report was from Amira Pharmaceuticals, describing the development and testing of AM966, a specific LPA_1_ antagonist. AM966 demonstrated a good pharmacokinetic profile following oral administration in mice and was shown to reduce lung tissue injury, vascular leakage, inflammation and fibrosis in a mouse model of bleomycin-induced lung fibrosis [129]. Bristol-Myers Squibb acquired Amira and continued the studies until bringing BMS-986020 (previously the LPA_1_ antagonist AM152 of Amira) to phase II clinical study in patients suffering from IPF (NCT01766817) [12]. Another LPA_1_ antagonist, SAR100842 from Sanofi, has been documented to be an effective antifibrotic agent. Indeed, orally administered in the Tsk1 mice (a model of systemic sclerosis), SAR100842 was able to inhibit myofibroblast differentiation, reduce skin collagen content and decrease hypodermal thickening to levels comparable to those obtained with the best available treatment (i.e., imatinib) [130]. In addition, SAR100842 prevented the LPA-induced myofibroblast differentiation of primary fibroblasts obtained from the dermis of patients with systemic sclerosis or from the lung of IPF patients [130]. Likewise, the LPA_1_ antagonist from Bristol-Myers Squibb, SAR100842, has been successfully tested in a phase II clinical study (NCT016551143) [131]. A step forward in clinical studies has been achieved by GLPG1690, a selective ATX inhibitor discovered by Galapagos, which is ready to be tested in two randomized placebo-controlled phase III trials in patients with IPF [132].

The above-mentioned clinical trials pave the way for future clinical studies aimed at testing the anti-ATX/LPA approach as a new therapy for cancer patients. Nevertheless, in view of the prosurvival, proangiogenic and profibrotic effects exerted by LPA on tumor and nontumor cells, the anti-ATX/LPA approach could also be useful as an adjuvant therapy for overcoming resistance to chemotherapy or to help inhibit angiogenesis or alleviate the side effects of radiotherapy-induced fibrosis. In addition, based on data showing that the LPA/LPA_5_ engagement functions as inhibitory signaling for the suppression of antigen-specific cytotoxic activity of CD8^+^ T cells, the anti-ATX/LPA approach could also offer the advantage of helping to overcome immunotherapy resistance.

Beyond the protumor effects summarized above, LPA may also be responsible for tumor-contrasting activities, as in the case of the LPA-induced stimulation of T cell endothelial transmigration capability, a process crucial in allowing T cell intratumor trafficking.

Therefore, to build safe and effective anticancer therapies based on the anti-ATX/LPA approach, it is worth considering the use of antagonist(s) capable of blocking the specific LPA receptor(s) responsible for the protumor effect(s), rather than blocking all ATX activity; this means that a future challenge will be to identify precisely which receptor mediates the protumor effect of LPA to inform the development of specific LPA-receptor antagonists.
